# Supplementary material for: Synthesizing a Water-Soluble Polymeric Nitrification Inhibitor with Novel Soil-Loosening Ability
Source: Polymers (Basel). 2023 Dec 29;16(1):107. doi: 10.3390/polym16010107 (PMC10780483; doi:10.3390/polym16010107)
Supplement: Supplementary file 1 [file polymers-16-00107-s001.zip › polymers-2742930-supplementary.pdf]

Supplementary Material

Synthesizing a Water-Soluble Polymeric Nitrification Inhibitor with Novel Soil-Loosening Ability

Yu Liu [a], Hui Gao [a, b]*, Shanshan Liu [a, b]*, Jinrong Li [c], Fangong Kong [a, b]

[a] State Key Laboratory of Biobased Material and Green Papermaking, Qilu University of Technology, Shandong Academy of Sciences, Jinan 250353, China

[b] Key Laboratory of Paper Science and Technology of Ministry of Education, Faculty of Light Industry, Qilu University of Technology, Shandong Academy of Sciences, Jinan, 250353, China

[c] School of Mechanical Engineering, Hebei University of Technology, Tianjin, 300401, China

* Corresponding author: **Hui Gao, Shanshan Liu**

E-mail: **gaohui1412@163.com, liushanshan8303@163.com**

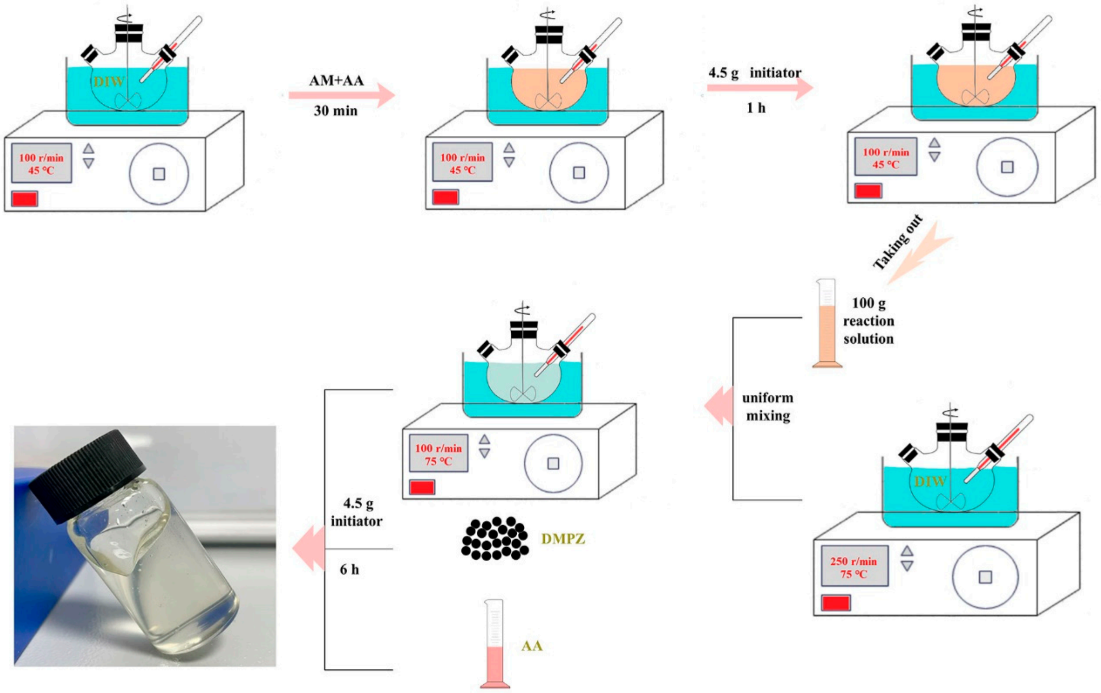

Figure S1. Fabrication processes of WSPNI.

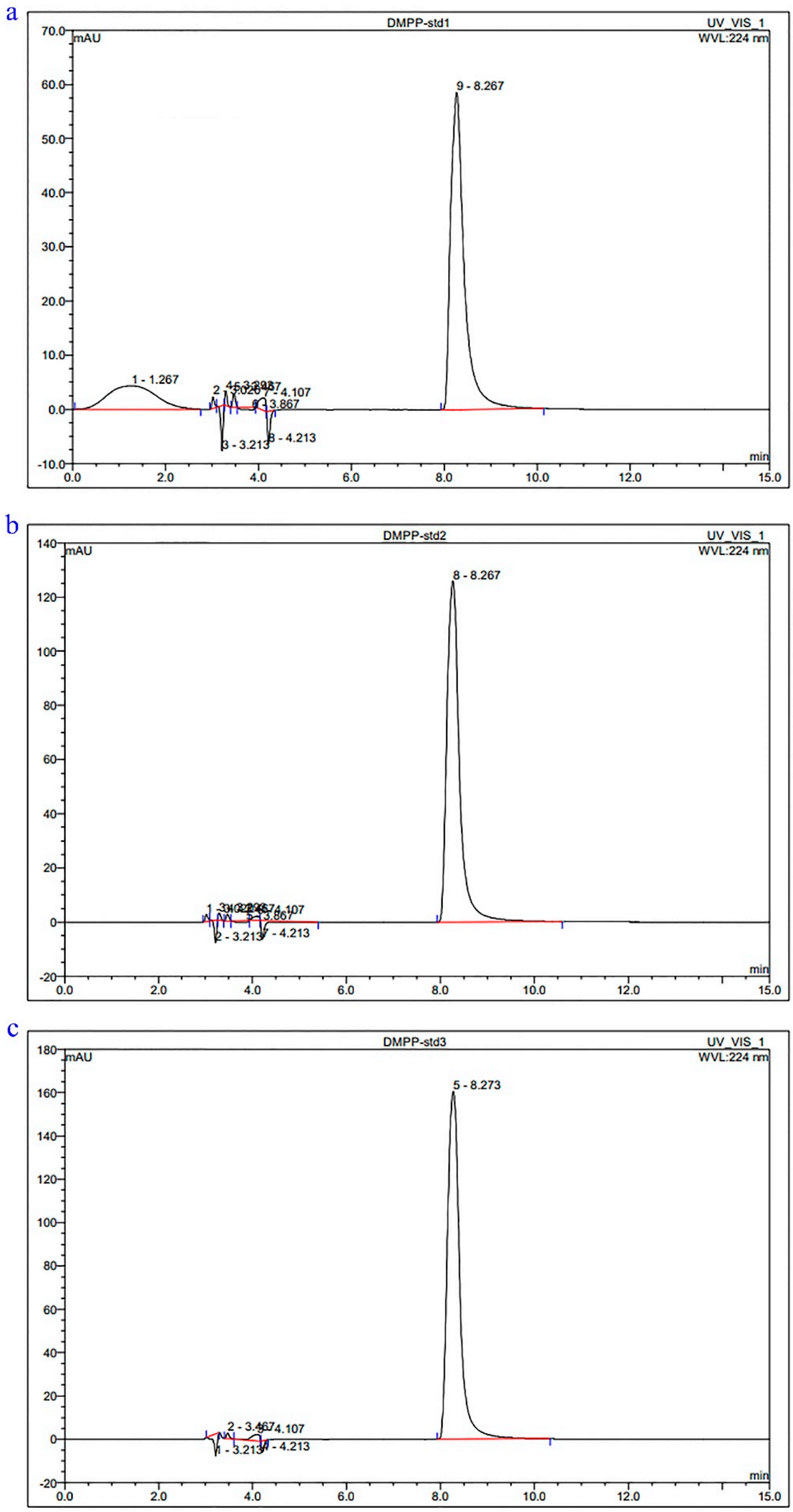

Figure S2. HPLC figures of DMPP standard sample with a concentration of (a) 51.60, (b) 102.04, and (c) 130.76 μg/mL.

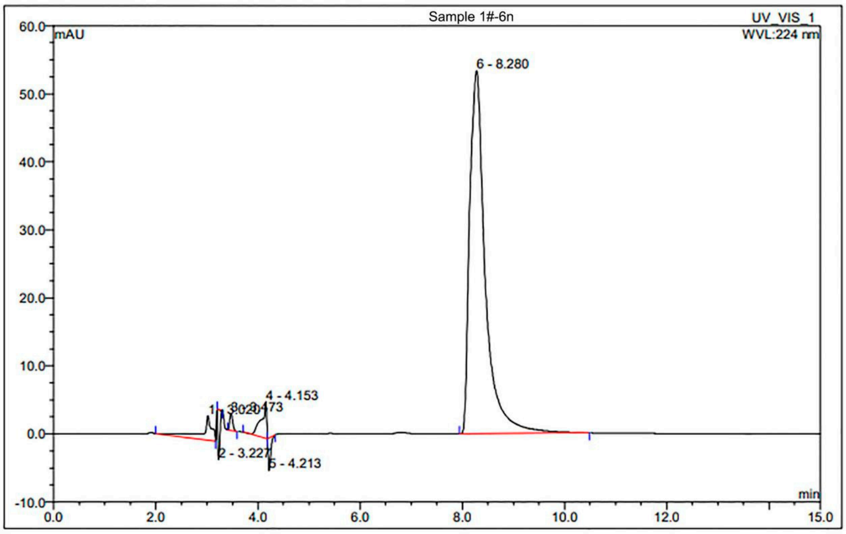

Figure S3. HPLC picture of WSPNI aqueous solution with a concentration of 49.60 μg/mL.

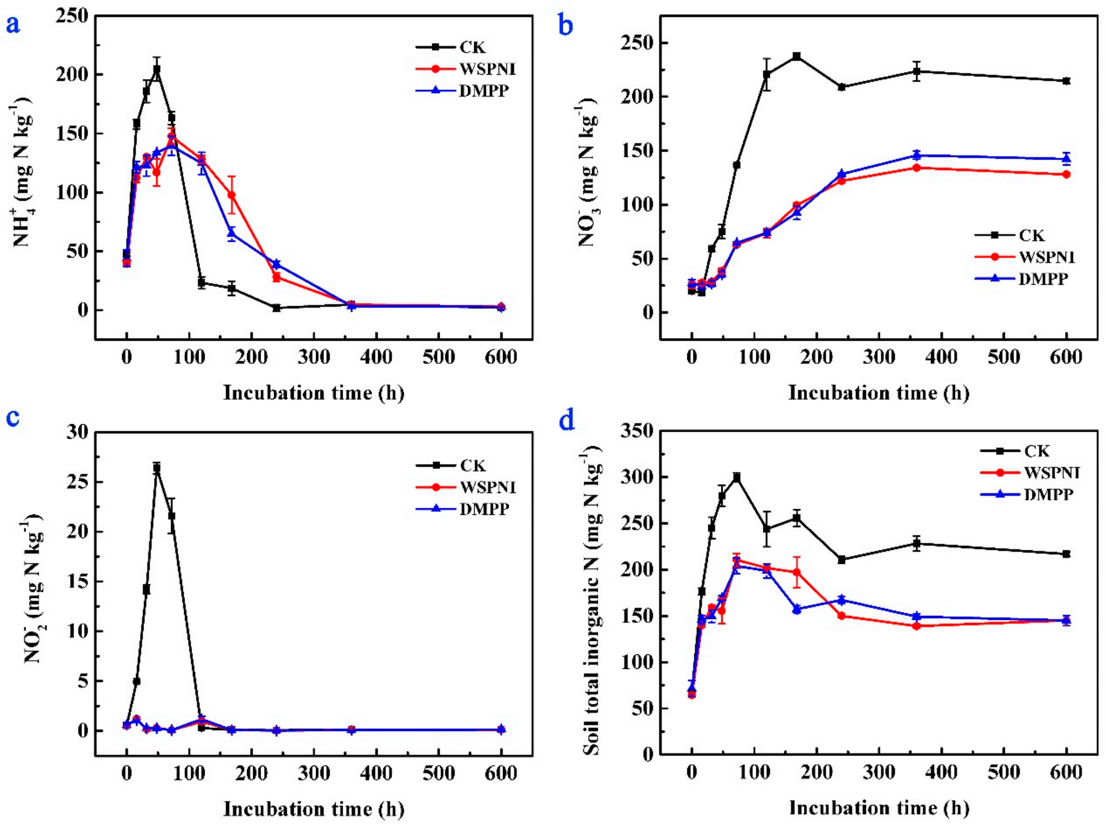

Figure S4. Changes of (a) NH_4^+-N concentration, (b) NO_3^--N concentration, (c) NO_2^--N accumulation amount and (d) total inorganic nitrogen amount in soil under different incubation times.

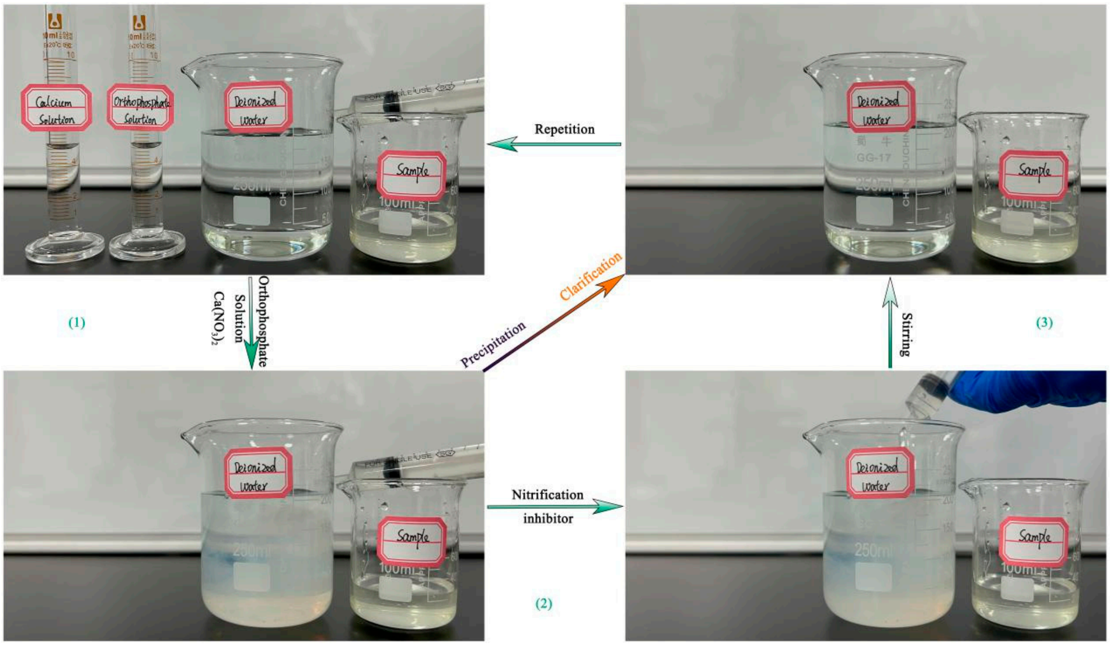

Figure S5. The phosphorous-solubilizing process of WSPNI.
